# Suppressing Structural Relaxation in Nanoscale Antimony to Enable Ultralow‐Drift Phase‐Change Memory Applications

**DOI:** 10.1002/advs.202301043

**Published:** 2023-06-28

**Authors:** Bin Chen, Xue‐Peng Wang, Fangying Jiao, Long Ning, Jiaen Huang, Jiatao Xie, Shengbai Zhang, Xian‐Bin Li, Feng Rao

**Affiliations:** ^1^ College of Materials Science and Engineering Shenzhen Key Laboratory of New Information Display and Storage Materials Shenzhen University Shenzhen 518060 China; ^2^ State Key Laboratory of Integrated Optoelectronics College of Electronic Science and Engineering Jilin University Changchun 130012 China; ^3^ Department of Physics Applied Physics, and Astronomy Rensselaer Polytechnic Institute Troy NY 12180 USA

**Keywords:** antimony, phase‐change memory, resistance drift, structural relaxation

## Abstract

Phase‐change random‐access memory (PCRAM) devices suffer from pronounced resistance drift originating from considerable structural relaxation of phase‐change materials (PCMs), which hinders current developments of high‐capacity memory and high‐parallelism computing that both need reliable multibit programming. This work realizes that compositional simplification and geometrical miniaturization of traditional GeSbTe‐like PCMs are feasible routes to suppress relaxation. While to date, the aging mechanisms of the simplest PCM, Sb, at nanoscale, have not yet been unveiled. Here, this work demonstrates that in an optimal thickness of only 4 nm, the thin Sb film can enable a precise multilevel programming with ultralow resistance drift coefficients, in a regime of ≈10^−4^–10^−3^. This advancement is mainly owed to the slightly changed Peierls distortion in Sb and the less‐distorted octahedral‐like atomic configurations across the Sb/SiO_2_ interfaces. This work highlights a new indispensable approach, interfacial regulation of nanoscale PCMs, for pursuing ultimately reliable resistance control in aggressively‐miniaturized PCRAM devices, to boost the storage and computing efficiencies substantially.

## Graphical abstract

The 4 nm‐thick monoatomic Sb film enables the ultralow resistance drift coefficient *v*, ranging from ≈10^−4^ to ≈10^−3^, which will benefit a further promotion in multibit programming accuracy to develop high‐capacity universal memory and high‐efficiency computing chips.

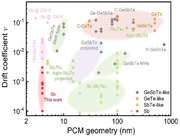

## 1 Introduction

Phase‐change random‐access memory (PCRAM), encoding digital information via reversible structure transformations between amorphous and crystalline phases of chalcogenide phase‐change materials (PCMs),^[^ ^1^, ^2^, ^3^, ^4^, ^5^, ^6^ ^]^ is the leading candidate for the developments of universal memory and neuromorphic computing chip to renovate classical von Neumann computing system.^[^ ^7^, ^8^, ^9^, ^10^ ^]^ High‐capacity memory and high‐parallelism computing both prefer multibit programming by each and every PCRAM device.^[^ ^11^, ^12^, ^13^ ^]^ Multiple resistance levels in a single PCRAM device are implemented by adjusting the amorphous‐to‐crystalline volume ratio under different external electrical stimuli.^[^ ^14^, ^15^ ^]^ The amorphous PCMs, for example, GeTe and GeSbTe, undergo structural relaxation, known as aging procedure, toward more equilibrium configurations with time,^[^ ^16^, ^17^ ^]^ rendering a steady increase in resistance magnitude at ambient temperatures.^[^ ^18^ ^]^ The drifted resistance states eventually blur the data resolution of intermediate resistance states, resulting in unavoidable decoding errors, which has become a bottleneck problem in memory and computing.^[^ ^5^, ^11^, ^19^ ^,^ ^20^ ^]^

**Figure 1:**
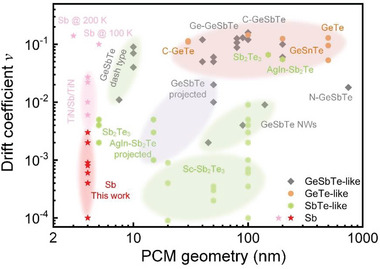
Strategies for reducing resistance drift. Resistance drift coefficient *v* versus PCM geometry (thickness, width, or diameter) relationship of diverse PCMs, including bulk films (in mushroom‐type devices) (orange area), nanowires (NWs), and slim films in dash‐type devices (green areas), as well as those in projected devices (purple area). As compared to the literature values (Figure S1, Supporting Information), the simplest PCM Sb (pink area) with only 4 nm thickness in this work enables the lowest *v*, in a range of ≈10^−4^–10^−3^. Note that the projected devices decouple device resistance READ‐out actions from WRITE/ERASE operations,^[^ ^18^ ^]^ therefore the low *v* of projected device does not reflect the intrinsic drift property of the PCM.

The aging phenomenon of PCMs can be characterized by measuring the resistance drift coefficient (*v*) within a certain period of time *t*, for example, ≈10^3^–10^5^ s, using the equation of *R*_t_ = *R*_0_ (*t*/*t*_0_)*^v^*, ^[^ ^18^ ^]^ where *R*_0_ and *R*_t_ is the initial and final resistance value measured at time *t*_0_ and *t*, respectively. “Bulk” GeTe‐ and GeSbTe‐like PCMs (tens to hundreds nm in thickness) exhibit pronounced resistance drift in the as‐deposited or melt‐quenched amorphous state with a quite large *v* of ≈0.11 (**Figure** **1** and Figure S1, Supporting Information),^[^ ^16^, ^17^, ^18^, ^19^ ^]^ whereas the drift of the crystalline state is only marginal. Reducing homopolar Ge‐Ge bonds and tetrahedral Ge motifs, as well as reinforced Peierls distortion, together form the driving forces of the noticeable structural relaxation of the vitreous phases.^[^ ^17^, ^21^ ^]^ Apparently, compositional simplification via eliminating Ge contents can facilitate the reduction of *v*, as witnessed in the bulk SbTe‐like PCMs with the *v* being ≈0.055 (AgIn doped Sb_2_Te)^[^ ^22^ ^]^ and ≈0.066 (Sb_2_Te_3_).^[^ ^23^ ^]^ Very recently, Sc doped Sb_2_Te_3_ films were found to enable ultralow *v* (≈10^−4^–10^−3^),^[^ ^24^ ^]^ owing to the enhanced rigidity across the medium‐to‐long range of the amorphous network and the reduced Peierls distortion in short range, both of which were attributed to the nanoscale inhomogeneity of Sc distribution. Another feasible way to decrease *v* is miniaturing the PCM geometry (or the active phase‐change volume in the device); for instance, GeSbTe nanowires of ≈45–140 nm in diameter can reduce the *v* to ≈0.002–0.009 level (Figure 1),^[^ ^25^ ^]^ because the much‐enlarged surface‐to‐volume ratio benefits a more efficient stress release in structural relaxation. Similarly, in dash‐type devices, the GeSbTe‐like film of ≈7.5 nm in width enables a tenfold smaller *v* of ≈0.011 (Ref. 26) as compared to the bulk counterparts. Further removing Ge component promotes more marked reductions in the *v*, ≈0.002–0.005,^[^ ^23^ ^]^ for the ≈5 nm‐thick Sb_2_Te_3_ nanolayers in the heterostructured architecture (Figure 1).

We note that the abundant lone‐pair electrons on Te atomic chains promote the creation of valence alternation pairs (VAPs),^[^ ^27^, ^28^, ^29^, ^30^ ^]^ that is, the localized charged defect states, in the energy bandgap of chalcogenide glasses. Either generation or recombination of the charged VAP defects can modify electronic band structure sensitively,^[^ ^28^, ^31^ ^]^ in consequence, removing Te element would probably relieve the relevant disturbance in conductance upon aging for the simplest PCM, that is, Sb, in its amorphous phase. Using the monatomic Sb as the PCM provides a promising route to tackle the reliability or fatigue issues when programming multicomponent compounds.^[^ ^24^ ^]^ However, bulk Sb film has no such glassy phases due to its spontaneous crystallization nature at room temperature,^[^ ^32^, ^33^ ^]^ which hinders non‐volatile storage in conventional mushroom‐type PCRAM devices. To acquire a stable amorphous phase, the Sb film has to be confined into sub 5 nm scale.^[^ ^32^, ^33^ ^]^ At low temperatures, for example, ≈100–200 K, the amorphous 5 nm‐thick Sb film sandwiched by SiO_2_ layers has a large *v* of ≈0.10–0.14,^[^ ^32^, ^34^ ^]^ whereas at room temperature, the 4 nm‐thick amorphized Sb sample clamped by TiN electrodes exhibits a very small *v* in the regime of ≈0.006–0.030 (Figure 1).^[^ ^33^ ^]^

Suppressing the resistance drift to an even lower level is technologically desired, thereby programming numerous but stabler bits in every single PCRAM cell will become possible, which can boost the computational capability and efficiency for resolving complicated artificial intelligence tasks, when a large‐scale neural network by dense arrays has to be employed.^[^ ^5^, ^11^, ^12^, ^13^ ^]^ Whether an even lower *v* can be accomplished by the Sb‐based PCRAM devices is still elusive, yet the aging mechanism of nanoscale Sb has not been touched in previous studies.^[^ ^32^, ^33^ ^]^ In general, at the reduced dimension interfacial effects should stand out and play a crucial role in affecting the kinetics of structural adjustment,^[^ ^35^, ^36^, ^37^ ^]^ including the relaxation process, while after all these years it has not been unveiled. In this work, by combining film and device characterizations with density functional theory (DFT) simulations, we reveal that the ultralow resistance drift (*v* ≈ 10^−4^–10^−3^, Figure 1) in the SiO_2_‐sandwiched ≈4 nm‐thick Sb films should originate from the slightly changed Peierls distortion in Sb and the less‐distorted octahedral‐like atomic motifs across the Sb/SiO_2_ interfaces. Our work sheds light on a new approach, that is, interfacial regulation of nanoscale PCMs, to tailor ultralow resistance drift of PCRAM cells for the design of multibit memory and accurate computing chips.

## 2 Results and Discussions

### 2.1 Structural Stability of Amorphous Phase

**Figure 2:**
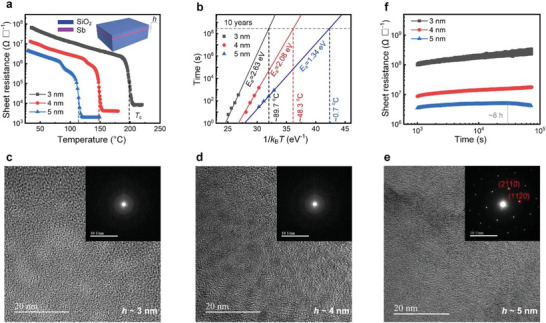
Structural stability of thin Sb films. a) Temperature dependence of the sheet resistance of SiO_2_‐sandwiched Sb films with the thickness *h* of 3, 4, and 5 nm (inset), at the same heating rate of 20 °C min^−1^. The crystallization temperature (*T*_c_) is indicated by the dashed line: ≈200, ≈150, and ≈115 °C for the 3, 4, and 5nm–thick sample, respectively. b) 10‐year data retention abilities for all the thin Sb samples. The data were fitted using the Arrhenius equation *t* = *A*exp(*E*_a_/*k*_B_ *T*), where *t* is the time to failure when the film sheet resistance of amorphous phase, at a certain isothermal heating temperature, falls to half of its initial magnitude; *A* is a proportionality constant, *E*_a_ is the activation energy of crystallization, and *k*_B_ is the Boltzmann constant. c–e) HRTEM image of 3, 4, and 5nm–thick Sb film after aging for ≈2 weeks, respectively. SAED pattern of each sample is shown in the corresponding inset. The aged 3 and 4nm–thick samples were still in amorphous phase, whereas the aged 5 nm‐thick one crystallized into hexagonal phase. f) Sheet resistance as a function of time for the as‐sputtered Sb samples (measured right after deposition), at room temperature and up to ≈7 × 10^4^ s. The 3 and 4nm–thick samples showed monotonically increasing resistance values throughout the measurements, while the 5 nm‐thick one started the resistance reducing process after ≈8 h aging.

To acquire stable amorphous Sb phase, we reduced the thickness *h* of the as‐deposited films to sub ≈5 nm (inset of **Figure** **2**a) to produce a stable amorphous phase. Temperature‐dependent electrical resistance measurements were performed for the SiO_2_‐sandwiched Sb films of 3, 4, and 5 nm in thickness upon in situ annealing at a heating rate of 20 °C min^−1^ (Figure 2a), since this technique is very sensitive to structural changes in PCMs.^[^ ^31^ ^]^ Thinner Sb film exhibited a higher sheet resistance at initial state, corresponding to the larger energy bandgap or stronger electron localization at around Fermi level (*E*_F_) of the amorphous semiconductor.^[^ ^2^, ^16^ ^]^ All the three amorphous Sb thin films transformed into the crystalline phase swiftly once reaching the crystallization temperature (*T*_c_), manifesting as a sharp reduction in sheet resistance. The higher *T*_c_ of the thinner Sb film, along with the larger activation energy of crystallization (*E*_a_), denotes the strengthened thermal stability of the amorphous phase. This is in line with 10‐year data retention ability shown in Figure 2b, where the thinner sample possesses a higher retention temperature for the amorphous data, that is, 89.7, 48.3, and 0.7 °C for the 3, 4, and 5 nm‐thick samples, respectively.

The amorphous nature of the 3 and 4 nm‐thick Sb films was also confirmed by high‐resolution transmission electron microscope (HRTEM) observation, because no obvious crystallites can be found in Figure 2c,d, and both selected area electron diffraction (SAED) patterns show dim halos, instead of sharp diffraction rings of crystals. Note that the HRTEM samples were aged at room temperature (≈27 °C) for ≈2 weeks before characterization, suggesting the strong anti‐recrystallization ability of the two amorphous samples. In contrast, the 5 nm‐thick sample transformed into crystalline with large hexagonal grains after the same aging period (Figure 2e). When monitoring sheet resistance of the 5 nm‐thick sample at room temperature, the gradually increasing trend ceased at ≈3 × 10^4^ s (≈8 h) (Figure 2f), following with a noticeable reduction (Figure S2, Supporting Information) caused by the spontaneous crystallization. Such an unstable amorphous phase will give rise to data loss in practical PCRAM use. By comparison, the other two thinner samples of stabler amorphous phase presented a steady increase in sheet resistance up to ≈7 × 10^4^ s (≈20 h) without any sign of decline (Figure 2f). It is noteworthy that the as‐deposited PCM films started relaxation immediately right after completion of deposition process,^[^ ^38^ ^]^ but it usually took about ≈1000 s (≈0.3 h) for us to get the capped film samples out of vacuum chamber and transfer onto the hot stage for resistance monitoring. Therefore, the starting point for all the sheet resistance measurements was set as 1000 s (≈0.3 h) in Figure 2f and Figure S2, Supporting Information.

### 2.2 Resistance Drift Performances

**Figure 3:**
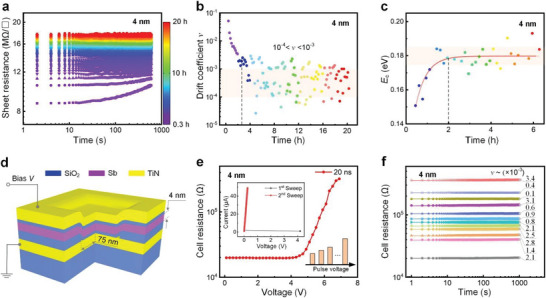
Resistance drift behavior of the 4 nm‐thick Sb film. a) Segmented curves of sheet resistance as a function of time for the 4 nm‐thick Sb films, measured at room temperature. Each curve has the equal period ≈600 s. The curve color indicates the drift time ranging from ≈1000 s up to ≈20 h. The sheet resistance increased steadily throughout the measurements, and at the initial stage (from first to fourth curve), the rising trend of sheet resistance was more obvious. b) The fitted resistance drift coefficient values (sub *v*) for each curve in (a), utilizing the equation *R*_t_ = *R*_0_ (*t*/*t*_0_)*^v^*, where *R*_0_ and *R*_t_ is the initial and final resistance value measured at time *t*_0_ and *t*, respectively. A clear drop of sub *v* values can be observed during the first ≈2 h as indicated by the dashed line, while afterward the sub *v* values mainly distribute in the range of ≈10^−4^–10^−3^. c) The calculated activation energy of conduction values of sub *E*_c_ for the first ≈6 h segmented curves in (a), showing a noticeable inflection at ≈2 h. d) Schematic of the 4 nm‐thick Sb‐based PCRAM device with the ≈150 × 150 nm^2^ contact area between SiO_2_/Sb/SiO_2_ stack and bottom TiN electrode. e) Progressive RESET operation curve of the 4 nm‐thick Sb‐based device under fixed electrical pulse width of 20 ns but increasing pulse magnitude (inset pulse waveforms), showing a continuous (gradual) climbing in cell resistance with voltage increment. Inset shows the current‐voltage curve of the 4 nm‐thick Sb‐based device, where the first sweep transformed the amorphous Sb into a highly conductive crystalline phase via threshold switching and Joule heating, then the second sweep displayed the linear ohmic relation. f) Temporal evolution of 12‐level resistance states achieved by iterative RESET operations in the 4 nm‐thick Sb‐based device, showing low resistance drift coefficients (≈1.0 × 10^−4^ < *ν* < ≈3.4 × 10^−3^) at room temperature. The pulse width was fixed at 20 ns, with the amplitude increasing from ≈4.8 V to ≈7.1 V.

Resistance drift is more severe for the thinner samples (Figure 2f): specifically, the drift amplitude is larger (approximately three times) in the 3 nm‐thick case, that is, from ≈10^8^ to ≈3 × 10^8^ Ω ⬜^−1^, while approximately twofold in the 4 nm‐thick one, that is., from ≈9 × 10^6^ to ≈1.8 × 10^7^ Ω ⬜^−1^. Considering the relatively stable amorphous phase accompanied with a milder resistance drift, the 4 nm‐thick scheme therefore became our focal point in subsequent studies of aging mechanisms and device performances. To gain a more precise fitting for the long‐term drift, we hereby divided the whole curve in Figure 2f into continuous segments, each containing an equal period of ≈600 s (**Figure** **3**a). By this means, one can clearly compare the drift amplitude in different aging stages, and conduct an easier and more accurate calibration for each sub *v* (Figure 3b), to survey the drift evolution more intuitively. We found that the drift behavior of the 4 nm‐thick sample was violent at the initial stage (starting point at ≈0.3 h), while it gradually became moderate until ≈2 h (Figure 3a,b). A clear drop in the sub *v* values was observed for the first ≈2 h duration of aging (Figure 3b). After that and till ≈20 h, the drift amplitude became rather limited (Figure 3a), when the sub *v* values majorly oscillated in a very small numerical range of ≈10^−4^–10^−3^ (Figure 3b).

The steady resistance increase of the aging PCMs owes to the annihilation of mid‐gap or band‐tail defect states, accompanied by a shifted electronic *E*_F_ toward the mid gap or a widened electronic energy bandgap (*E*_g_).^[^ ^17^, ^18^, ^21^ ^]^ The activation energy of conduction (*E*_c_) that is approximately half of the *E*_g_ for many glassy chalcogenides should also be enlarged as aging progresses.^[^ ^27^, ^31^ ^]^ The analogous drift behavior of the 4 nm‐thick Sb film may be attributed to the similar bandgap opening mechanism. Note that a physically relevant measurement of the *E*_c_ can only be taken during a temperature cooling ramp^[^ ^22^ ^]^ (Figure S3a, Supporting Information), when the thermally activated drift is strongly restrained. We thus conducted the sort of measurements to collect sub *E*_c_ values at different aging stages (Figure S3b, Supporting Information). The sub *E*_c_ value slightly rises from ≈0.15 eV initially to ≈0.18 eV at ≈2 h, and afterward it oscillates around this saturated value till ≈6 h (Figure 3c). The evolution manners of sub *v* and sub *E*_c_ coincide with each other, both inflecting at ≈2 h. Here, if the *E*_g_ of the aged 4 nm‐thick amorphous Sb film is about ≈2*E*_c_, that is, ≈0.36 eV, whereas its crystalline counterpart should be a metallic conductor with almost zero bandgap, the distinct conduction abilities between the two phases may permit a certain number of intermediate resistance states when programming its PCRAM device.

We then incorporated the 4 nm‐thick amorphous Sb film into a PCRAM device (Figure 3d) to verify this hypothesis. The thin Sb film was sandwiched by ≈1.5 nm‐thick SiO_2_ films. Note the ultrathin SiO_2_ nanolayers cannot block electrical conduction, as the multilayered SiO_2_/PCM would still execute reversible phase transitions in devices.^[^ ^39^ ^]^ In addition, the glassy SiO_2_ nanolayers can add an invariable and large series resistance to the thin Sb resistor, which may help to enhance the anti‐drift ability of the high‐resistance states of the device. It usually took several days to fabricate the PCRAM device, meanwhile the amorphous Sb film in the device had already been deeply aged and arrived at a rather equilibrium state before the electrical characterization. Therefore, it is practically impossible to detect a device with minimized aging, and one could envisage a quite low drift performance for the as‐fabricated Sb‐based device. We first transformed the amorphous Sb film into crystalline (SET operation) via DC current‐voltage sweeping, by which a typical threshold switching process occurred (inset of Figure 3e). Then we amorphized the crystalline film into the melt‐quenched phase (RESET operation) by employing 20 ns‐width voltage pulses with increasing magnitude (Figure 3e), where the device resistance progressively increased when the voltage exceeded ≈5 V, and finally saturated at ≈7 V, which is in stark contrast with the sudden jump of resistance observed in other devices using bulk PCMs.^[^ ^40^ ^]^ This sluggish RESET behavior could be ascribed to the modulation of amorphous/crystalline ratio in the special device geometry, similar to our previous Sb‐based PCRAM device,^[^ ^33^ ^]^ which is good for catching intermediate states that requests progressive (iterative) amorphization.^[^ ^15^, ^23^ ^]^ We therefore carried out iterative RESET programming on the 4 nm‐thick Sb device, achieving 12 resistance levels with ultralow drift *v* values (Figure 1) ranging from ≈0.0001 to ≈0.0030 (Figure 3f). Each resistance level was immediately monitored after programming, where the time interval between the RESET and successive READ operations was only several ms.

### 2.3 Atomistic Models and Peierls Distortion

**Figure 4:**
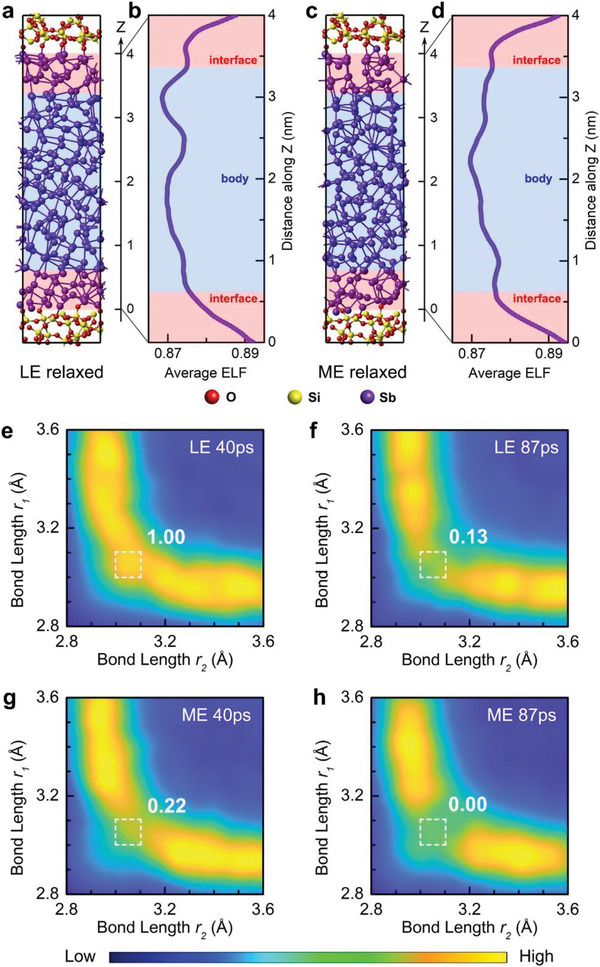
Amorphous thin Sb film models and Peierls distortion evolution. a,c) Amorphous LE and ME models of 4 nm‐thick Sb film sandwiched by glassy SiO_2_ dielectrics after annealing at 300 K for 87 ps, respectively. Bond‐length cutoff is 3.2 Å. b,d) Average ELF value along the thickness *Z* direction of the 4 nm‐thick Sb part in the (a) and (c) model, respectively. The interface (pink) regions in both models possess relatively higher average ELF values (≈0.872–0.895) than those of the body (blue) regions. e,f) The degree of Peierls distortion evaluated by angular‐limited three‐body correlation (ALTBC) analysis of Sb—Sb bonds for the 300 K‐annealed amorphous LE Sb model at initial (39.25–40.75 ps) and relaxed (86.25–87.75 ps) stage,^[^ ^45^, ^46^ ^]^ respectively, illustrating the probability of having a bond of length *r*_1_ almost aligned with a bond of length *r*_2_ (angular deviations smaller than 30°). g,h) The ALTBC pattern for the amorphous ME Sb model at initial and relaxed stage, respectively. The white numbers are the normalized average intensities in the representative bond length range of 3.0 Å < *r*_1_ < 3.1 Å and 3.0 Å < *r*_2_ < 3.1 Å as pointed out by the white dashed squares.

It is interesting to note that both the deeply‐aged film sample and the melt‐quenched device exhibited ultralow‐drift performances with the well‐matched *v* of ≈10^−4^–10^−3^ (Figure 3b,f), which is in contrast with the much larger *v* (≈10^−3^–10^−1^) for the as‐sputtered film sample at the initial ≈2 h aging stage. This discrepancy probably stems from different (less‐ or more‐equilibrium) initial microstructures^[^ ^41^ ^]^ of the two amorphous phases, leading to the divergent relaxation paths subsequently.^[^ ^42^, ^43^ ^]^ We then performed DFT‐based molecular dynamics (DFMD) simulations, generating 4 nm‐thick less‐equilibrium (LE) and more‐equilibrium (ME) amorphous Sb layers sandwiched by glassy SiO_2_ dielectrics (**Figure** **4**a,c), signifying the unrelaxed high‐energy and relaxed low‐energy phases before and after aging, respectively. The LE model was created by directly quenching the molten Sb from 1000 to 300 K to avoid any significant relaxation, emulating the highly energetic and non‐equilibrium sputtering deposition process.^[^ ^41^ ^]^ While the ME model was obtained via quenching the melt with a finite cooling rate of 29 K ps^−1^ (see Experimental Section for details). Both models were further equilibrated at 300 K for ≈90 ps to reach low‐energy state, during when the LE‐Sb always exhibited a slightly larger system energy than that of the ME‐Sb (Figure S4, Supporting Information).

We first compared the atomistic structures of the 300 K‐annealed LE and ME models at 87 ps (Figure 4a,c). Their local structures are quite similar in general, including the coordination numbers (CNs) and bond‐angle distributions (BADs) (Figure S5, Supporting Information). Both models have some heteropolar chemical bonds, that is, Sb—O or Sb—Si, across the Sb/SiO_2_ interfaces. Another interesting feature nearby the interfaces is that electrons on homopolar Sb—Sb bonds turn to be more localized, as the average electron localization function (ELF) values are noticeably larger (>≈0.872) than those for the internal Sb parts (Figure 4b,d). We thereby defined the interface and body regions according to the large/small ELF boundaries. The depth (≈0.6 nm) of the interface region in both models did not alter considerably during simulations. Similar interface depth is observed for 3 nm‐thick Sb (Figure S6, Supporting Information). Due to the interface‐proximity effect, the interface regions constitute stronger covalent bonds, deviating more from the metavalent bonding scheme of crystalline Sb lattice,^[^ ^44^ ^]^ which would help to stabilize a disordered network on 2D scale,^[^ ^37^ ^]^ against spontaneous recrystallization. As a result, scaling down the Sb film thickness effectively enables the stabilization amorphous phase (Figure 2b).

We then examined Peierls distortion for Sb—Sb bonds in both models, which describes a bonding hierarchy of long‐short bonding pairs having a ≈180° bonding angle.^[^ ^17^, ^44^, ^45^ ^]^ The larger discrepancy between bonds in a pair, the more significant Peierls distortion, and vice versa. One may notice that the central distribution for the bond length‐splitting ratio (*r*_2_/*r*_1_) between 1.00 (*r*_2_ = *r*_1_ ≈ 3.05 Å) and ≈1.07 (*r*_2_ ≈ 3.20 Å and *r*_1_ ≈ 3.00 Å), in the LE model, decreases dramatically upon relaxation from 40 to 87 ps. On the contrary, the ME model has a rather smaller distribution for 1.00 < *r*_2_/*r*_1_ < ≈1.07, and the decrease from 40 to 87 ps is not so significant either. This can be further quantitively confirmed by the normalized average intensities in the representative bond length range of 3.0 Å < *r*_1_ < 3.1 Å and 3.0 Å < *r*_2_ < 3.1 Å. As displayed by the bonded white numbers in Figure 4e–h, the normalized intensity decreases dramatically (0.87) upon relaxation from 40 to 87 ps in the LE model. On the contrary, the difference in the ME model is only 0.22 before and after relaxation. The comparison denotes that Peierls distortion continues to reinforce when Sb glass is aging, prominently for the more unstable LE model, whereas becoming quite marginally for deeply relaxed ME case. Strengthening Peierls distortion can help to localize electrons on the chemical bonds, which may open a pseudo‐bandgap,^[^ ^46^, ^47^ ^]^ giving rise to the larger resistivity. Once the evolution of Peierls distortion becomes stagnant, the resistance drift may be substantially alleviated. We also found that both models have the bigger Peierls distortion on the interface than in the body part (Figure S7, Supporting Information). Hence, because of the enhanced interface‐to‐body ratio, the thinner Sb sample ought to possess a bigger contrast in Peierls distortion evolution, which may be responsible for the larger drift in the 3 nm‐thick film and device (Figure S8, Supporting Information).

### 2.4 Density of States and Heteropolar Bonds

**Figure 5:**
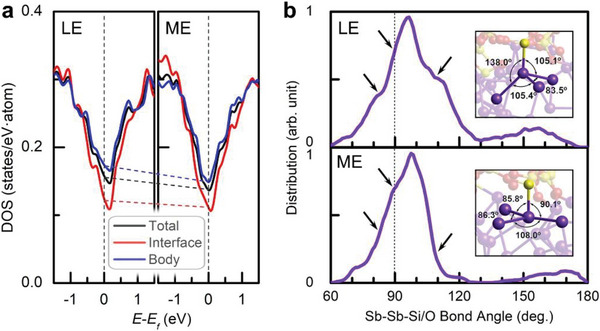
Interfacial driving force on structural relaxation. a) Electronic density of states (DOS) of the 300 K‐annealed LE and ME Sb model at 87 ps. The *E*_F_ levels computed at 0 K for the two models are marked as vertical dashed lines. Respective contribution by the body and interface regions is shown as the curve in corresponding color. The ME phase has deeper state distributions on *E*_F_ than those of the LE phase, as marked by the dashed lines, which suggests a widened *E*_g_ with fewer mid gap states when the LE phase relaxes toward the ME phase, in which the interface parts play the major role in deepening the state valley distribution. b) BADs of the Sb‐centered motifs with heteropolar Sb—Si/O bonds, in the relaxed LE and ME models, respectively. The striking differences, that is, shoulder peak versus steeper descent, between the two BADs are indicated by the arrows. Insets show the representative atomic motifs in two models with typical bonding angles being labeled. Bond‐length cutoff is 3.2 Å.

Next, we compared the electronic structures via calculating electronic density of states (DOS) of both 300 K‐annealed models at 87 ps (**Figure** **5**a), and separated the contributions by the body and interface parts. Note that the DFT description of the electronic exchange–correlation interaction by using generalized gradient approximation (GGA) usually underestimates the bandgap values.^[^ ^48^ ^]^ Defect states may reside on the band tail or in the mid gap, causing a seemingly continuous state distribution over the shallow gap.^[^ ^49^, ^50^ ^]^ Yet, one can still discern the differences in DOS distributions at the *E*_F_ of both models. As compared to the LE phase, the ME phase has a deeper total state‐distribution valley on *E*_F_, since its interface‐ and body‐contributed states on *E*_F_ both become fewer (Figure 5a), which indicates a widened *E*_g_ or reduced mid‐gap defects after aging. Hence, when a LE phase relaxes toward energetically more stable state, the film resistivity rises. This is in line with the obvious drift during the first 2‐h aging of the as‐sputtered Sb film (Figure 3a,b). Once reaching a rather equilibrium state, steady electrical conduction can be observed (Figure 3a,f).

Interestingly, the interface (body) contributes a relatively deep (shallow) state distribution on *E*_F_, proving the fact that the less‐conductive interfaces dominate the electrical conduction through the thin film. Furthermore, within the interfaces, the heteropolar (Sb—Si/O) bonds render a deeper state distribution than that by the homopolar (Sb—Sb) bonds (Figure S9, Supporting Information), which means the heteropolar bonds can affect the resistance drift behavior more profoundly. We then zoomed in the interfaces to inspect the atomic motifs containing the heteropolar bonds. Such motifs in both models (at 87 ps) show a strong BAD peaking at ≈100° (Figure 5b). For the motifs in the LE phase, there are two noticeable shoulder peaks at ≈80°–85° and ≈105°–115°, and a sharp decline at 90°, in the BAD (upper panel of Figure 5b). On the contrary, the BAD of the motifs in the ME phase only shows an obvious shoulder peak at 90°, and steep drops at other two corresponding regimes (lower panel of Figure 5b). The close to 90° and 109.5° bond angles favors octahedral and tetrahedral configurations, respectively. Hence, we observed the more octahedral‐like motifs in the ME phase (inset in lower panel), whereas the more distorted/tetrahedral‐like motifs in the LE phase (inset in upper panel). This comparison suggests that the Sb thin film needs to adjust its outer interfaces toward the less‐distorted octahedral‐like (for example via reducing the non‐*p*‐bonding tetrahedral motif)^[^ ^17^ ^]^ configurations upon relaxation to stabilize electrical conduction eventually.

## 3 Conclusion

We demonstrated that thinning the Sb film to sub ≈5 nm can strengthen the amorphous phase for non‐volatile phase‐change storage and computation. At this 2D scale, interfacial effect prevails in modulating electrical conduction throughout the thin film, because nearby the Sb/SiO_2_ interfaces, electrons become more localized and meanwhile Peierls distortion can be reinforced, together facilitating an increasing (drifting) resistance upon aging. With the optimal thickness of 4 nm, the deeply‐aged Sb thin film and the corresponding device accomplished ultralow drift with the *v* ranging from ≈0.0001 to ≈0.0030, while the as‐sputtered film displayed nearly 2 orders of magnitude larger *v* in the first 2‐h aging. We ascribed such discrepancies to the different energetical equilibrium states of the amorphous phases when initiating aging process. The less‐equilibrium Sb phase, after a long‐term relaxation, would evolve toward the more‐equilibrium form, via freezing the evolution of internal Peierls distortion and constructing less‐distorted octahedral‐like configurations on the interfaces, thus ensuring an almost invariant electrical conduction throughout the aging procedure. Our work explains the aging mechanisms of Sb thin film and sheds light on how to pursue high‐reliability multibit control of nano‐confined PCMs in the PCRAM devices, which can promote the developments of high‐capacity storage and high‐accuracy computing applications in big data era.

## 4 Experimental Section

### Film Preparation and Characterization

Sb thin film was prepared by sputtering Sb target (with purity of 99.999%) via physical vapor deposition (PVD) under ultrahigh vacuum with a base pressure of <≈2 × 10^−7^ Torr. The deposition rate was controlled to be ≈0.6 nm min^−1^. Atomic force microscope was then employed to characterize the surface morphology and thickness of the Sb and SiO_2_ films. The morphology and crystallinity were further confirmed by HRTEM (JEOL JEM‐2100F) at high tension of 200 kV. The sheet resistance of the thin Sb film was studied using a Linkam HFS600E‐PB4 hot stage with a temperature accuracy of 0.1 K. SiO_2_ capping layer (≈10 nm in thickness) was in situ grown on top of the Sb film inside the vacuum chamber to avoid oxidation for TEM and thermal measurements.

### Device Fabrication and Electrical Measurements

The PCRAM device was fabricated on a silicon substrate with 300 nm‐thick thermally oxidized SiO_2_ layer which served as thermal and electrical insulation. Then ≈30 nm‐thick TiN layer was deposited by sputtering Ti target (with purity of 99.99%) with controlled deposition rate of ≈1.7 nm min^−1^ in PVD. On top of the TiN bottom electrode layer, the cell structure was developed using electron beam lithography (EBL) patterning, following the ≈20 nm‐thick SiO_2_ deposition in PVD and lift‐off process. Then a via hole with the dimension of 150 × 150 × 20 nm^3^ was formed, surrounded by the dielectric SiO_2_ wall. Another EBL patterning process was required for the Sb film and top TiN electrode. ≈1.5 nm‐thick SiO_2_, ≈4 nm‐thick Sb, and ≈1.5 nm‐thick SiO_2_ layers were then sequentially deposited. To precisely control the thickness of the SiO_2_ layers, very low deposition rate of SiO_2_ (≈0.6 nm min^−1^) was adopted by lowering the deposition power. Then ≈30 nm‐thick TiN was in situ grown in the PVD as top electrode. The excess material was then removed by lift‐off technique. The electrical measurements for the PCRAM were performed by using the Keithley 2400C source meter (measuring device resistance) and the Tektronix AWG5202 pulse generator (generating nanoseconds voltage pulses).

### DFT Calculations

The DFMD simulations were performed in the Vienna Ab initio Simulation Package (VASP) code. The electron‐ion interaction was described by the projector augmented wave pseudopotential^[^ ^51^ ^]^ and the electronic exchange–correlation interaction was described by the GGA with the Perdew–Burke–Ernzerhof functional.^[^ ^52^ ^]^ The NVT canonical ensemble and Nosé thermostat were employed to control the temperature.^[^ ^53^ ^]^ An energy cutoff of 300 eV and the K‐point set of 2 × 2 × 1 were employed for the DFMD. This model has orthorhombic lattice parameters with *a* = *b* = 12.2 Å and *c* = 51.459 Å. This model used ≈10 Å‐thick amorphous SiO_2_ layers with a density of 2.2 g cm^−3^ sandwiching the 4 nm‐thick Sb film with a density of 6.69 g cm^−3^ to mimic the experimental situation. The model contains 293 atoms including 33 Si, 66 O, and 194 Sb. To obtain amorphous Sb/SiO_2_ interface at nanoscale, first, the area of Sb part was locked, meanwhile the SiO_2_ part was melted at 3000 K for 6 ps, and then quenched to 2000 K. After maintaining at 2000 K for 6 ps, the SiO_2_ part was quenched to 300 K, and further relaxed at 300 K for 6 ps to obtain the amorphous phase. Second, the amorphous SiO_2_ part was locked when the Sb part underwent melting process at 3000 K for 6 ps, followed by another melting duration at 1000 K for 6 ps. The amorphous LE model was created by directly placing the 1000 K‐melted Sb at 300 K, while the ME model was obtained by continuously quenching the 1000‐K melted Sb to 300 K with a cooling rate of 29 K ps^−1^. Both the LE and ME Sb models were further relaxed at 300 K for ≈90 ps for analyzing the atomic, bonding, and electronic properties.

## Conflict of Interest

The authors declare no conflict of interest.

## Author Contributions

B.C. and X.‐P.W. contributed equally to this work. B.C., F.Y.J., L.N., J.T.X. and J.E.H. fabricated the Sb thin film and PCRAM device samples and carried out microstructural characterizations and electrical measurements. X.‐P.W. performed DFT and DFMD simulations. F.R. and X.‐B.L. wrote the paper with contributions from S.B.Z., B.C., and X.–P.W. All authors discussed the results and commented on the manuscript.

## Supporting information

Supporting Information

## Data Availability

The data that support the findings of this study are available from the corresponding author upon reasonable request.
