# Supplementary material for: Suppressing Structural Relaxation in Nanoscale Antimony to Enable Ultralow‐Drift Phase‐Change Memory Applications
Source: Adv Sci (Weinh). 2023 Jun 28;10(25):2301043. doi: 10.1002/advs.202301043 (PMC10477879; doi:10.1002/advs.202301043)
Supplement: Supplementary file 1 — Supporting Information [file ADVS-10-2301043-s001.pdf]

ADVANCED SCIENCE
Open Access

Supporting Information

for *Adv. Sci.*, DOI 10.1002/advs.202301043

Suppressing Structural Relaxation in Nanoscale Antimony to Enable Ultralow-Drift Phase-Change Memory Applications

Bin Chen, Xue-Peng Wang, Fangying Jiao, Long Ning, Jiaen Huang, Jiatao Xie, Shengbai Zhang, Xian-Bin Li and Feng Rao**

Supporting Information for

Suppressing structural relaxation in nanoscale antimony to enable ultralow-drift phase-change memory applications

Bin Chen[1*], Xue-Peng Wang[1,2*], Fangying Jiao[1], Long Ning[1], Jiaen Huang[1], Jiatao Xie[1], Shengbai Zhang[3], Xian-Bin Li[2†], Feng Rao[1†]

[1]College of Materials Science and Engineering, Shenzhen Key Laboratory of New Information Display and Storage Materials, Shenzhen University, Shenzhen 518060, China.

[2]State Key Laboratory of Integrated Optoelectronics, College of Electronic Science and Engineering, Jilin University, Changchun 130012, China.

[3]Department of Physics, Applied Physics, and Astronomy, Rensselaer Polytechnic Institute, Troy, NY 12180, USA.

*These authors contributed equally to this work.

†Corresponding author. E-mail: fengrao@szu.edu.cn, lixianbin@jlu.edu.cn.

This PDF file includes:

1. (Figure S1) A comparison of resistance drift coefficients of diverse PCMs with different geometries.
2. (Figure S2) Resistance drift behavior of the 5 nm-thick Sb film.
3. (Figure S3) Measurements for the activation energy of conduction of the 4 nm-thick Sb film.
4. (Figure S4) Energy evolutions of the less-equilibrium (LE) and more-equilibrium (ME) 4 nm-thick amorphous Sb models annealed at 300 K.
5. (Figure S5) Coordination number (CN) distribution and Bond angle distribution (BAD) of the LE and ME models of Sb at 87 ps.
6. (Figure S6) Amorphous 3-nm thick ME models and corresponding ELF distributions.
7. (Figure S7) Peierls distortion for the body and interface parts in the two 4 nm-thick amorphous models.
8. (Figure S8) Larger resistance drift of the 3 nm-thick Sb films.
9. (Figure S9) Partial electronic density of states (DOS) contributed by heteropolar and homopolar bonds on the interfaces.

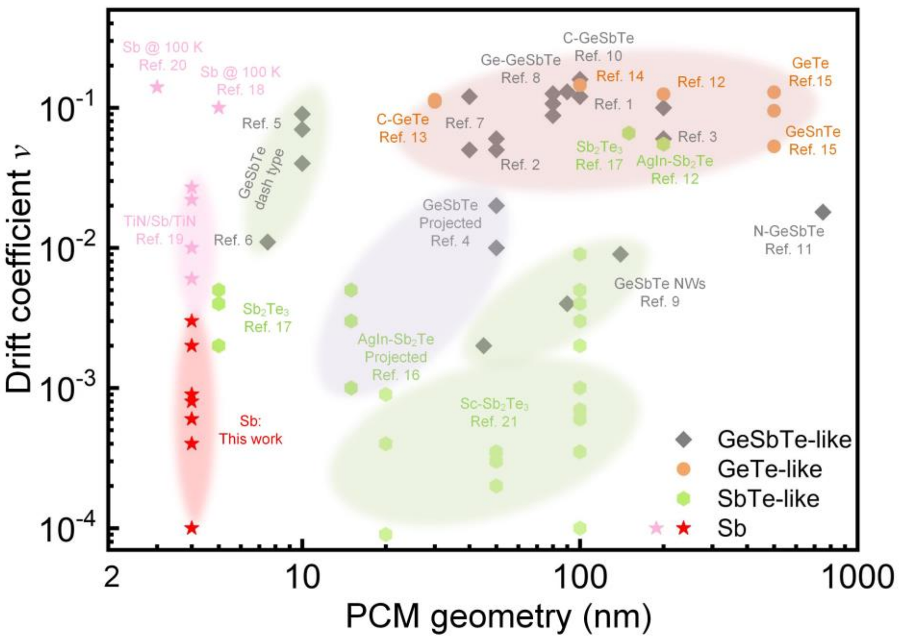

Figure S1. A comparison of resistance drift coefficients of diverse PCMs with different geometries. The distribution of resistance drift coefficient (v) for GeSbTe-like, GeTe-like, and SbTe-like PCMs from literature, as well as Sb thin film in this work. The corresponding references are detailed in this figure.

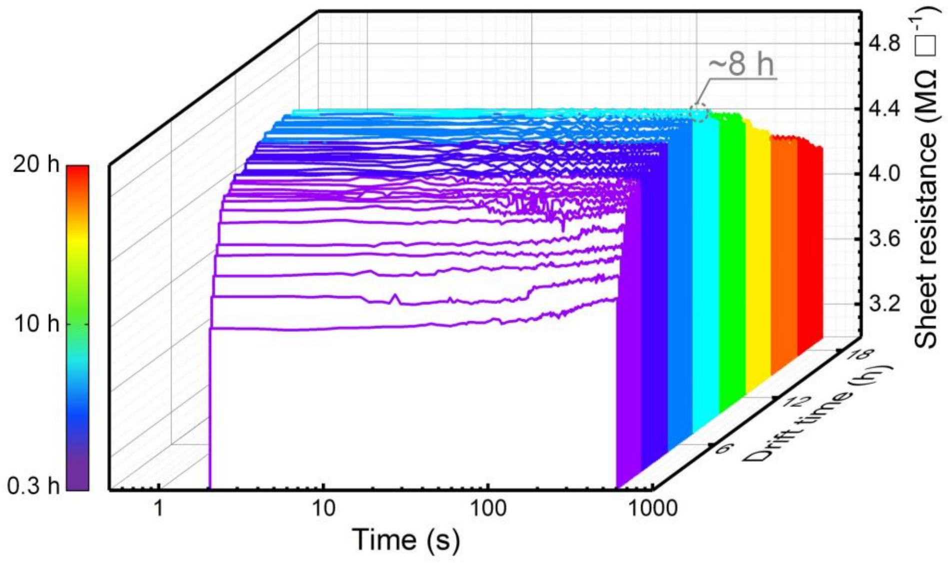

Figure S2. Resistance drift behavior of the 5 nm-thick Sb film. Sheet resistance as a function of time for the 5 nm-thick Sb film, measured at room temperature. The whole measured curve of nearly ~20 h duration is divided into multiple segments, each containing ~600 s. The sheet resistance of the 5 nm-thick Sb film increased with time steadily within the first ~8 h, whereas afterwards the sheet resistance continuously decreased due to the progressive crystallization.

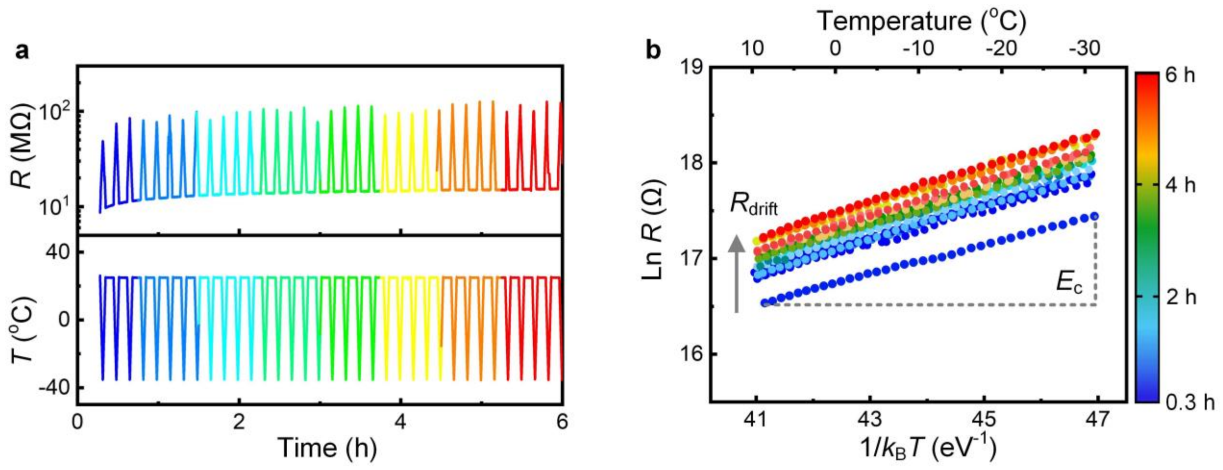

Figure S3. Measurements for the activation energy of conduction (E_c) of the 4 nm-thick Sb film. (a) The cooling and heating cycles (lower panel) and the corresponding sheet resistance of the 4 nm-thick Sb film (upper panel). Repetitive cooling and heating were conducted to the thin Sb sample in the temperature range of -35 to 25℃, for a whole duration about ~6 h. The sheet resistance of the sample was real-time monitored. (b) Arrhenius plot of the measured sheet resistance data. For each temperature dip in (a), once the measured resistance magnitudes and the changing trend became reliable in the regime from ~10 to ~-30℃, a single straight line was obtained, which was considered as a proper method to deduce the E_c value.[12] The line color indicates the drift time ranging from ~0.3 h up to ~6 h. The sheet resistance magnitude of the straight lines continuously increases upon drifting. Here we show some representative lines that were measured at different aging periods. The E_c is derived via fitting each straight line, utilizing the equation $R(T, t) = R^* \mathrm{e}^{\frac{E_c(T,t)}{k_B T}}$, with R^* the pre-factor, T the temperature, t the time, and k_B the Boltzmann constant, respectively.

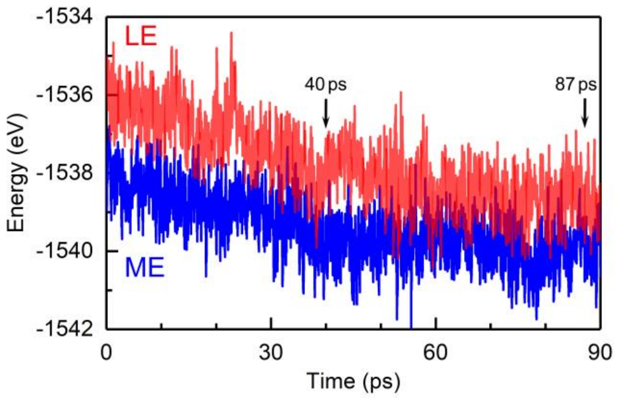

Figure S4. Energy evolutions of the less-equilibrium (LE) and more-equilibrium (ME) 4 nm-thick amorphous Sb models annealed at 300 K. The system energy of both models decreased noticeably until ~40 ps, and afterwards became relatively stable. We thus termed the ~40 ps and ~87 ps structures as the initial and relaxed states, respectively.

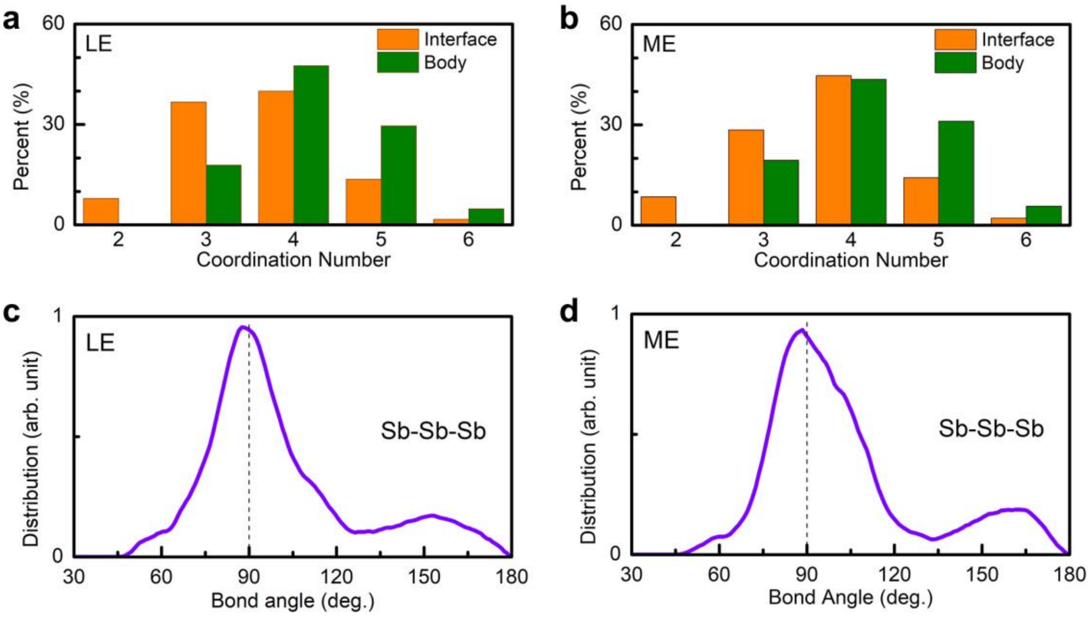

Figure S5. Coordination number (CN) distribution of the (a) LE and (b) ME model at 87 ps, respectively. The CN distribution is separated into two parts by the interface and body counts. Bond angle distribution (BAD) of the (c) LE and (d) ME models at 87 ps.

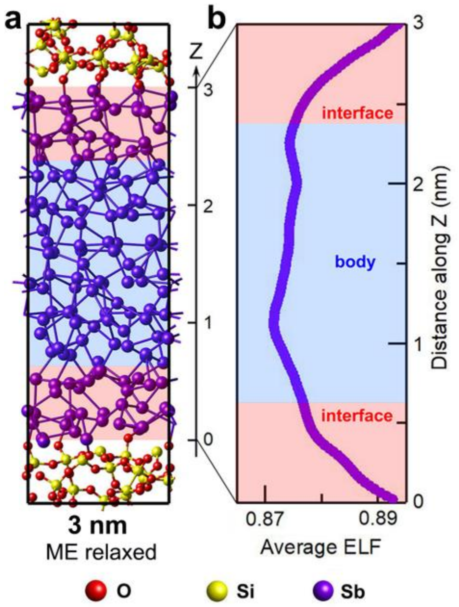

Figure S6. (a) Amorphous ME models of 3 nm-thick Sb film sandwiched by glassy SiO_2 dielectrics after annealing at 300 K for 87 ps. (b) Average ELF value along the thickness *Z* direction of the Sb part in the (a) model.

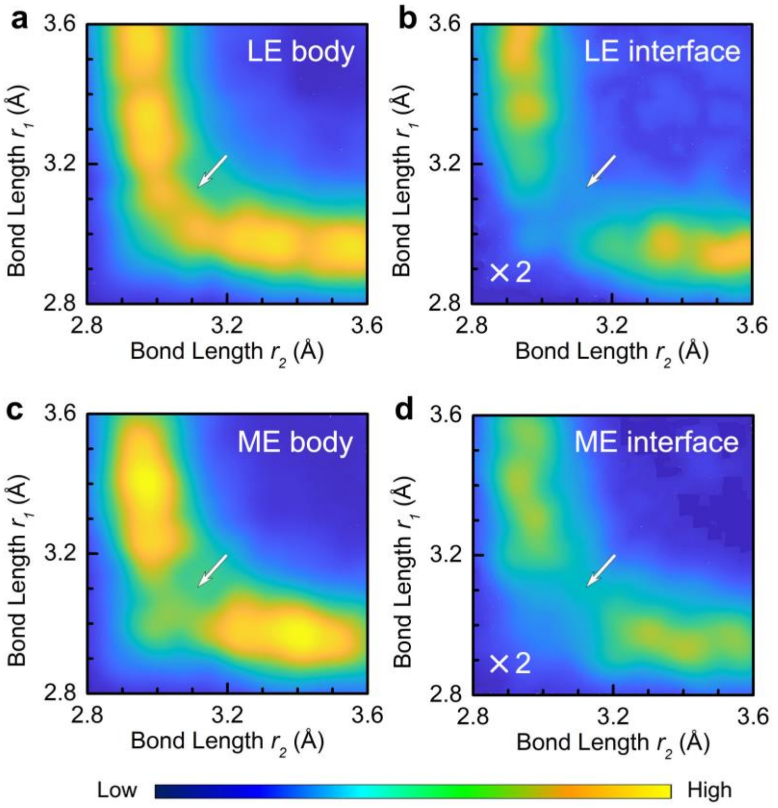

Figure S7. Peierls distortion for the body and interface parts in the two 4 nm-thick amorphous models. (a) and (b) The ALTBC pattern for the body part and the interface part in the LE model at 87 ps, respectively. (c) and (d) The ALTBC pattern for the body part and the interface part in ME model at 87 ps, respectively. In both models, the Peierls distortion is stronger on the interface than in the body, because the count distribution at around r_2 / r_1 ≈ 1.00-1.07 is larger/marginal for the interface/body part, as indicated by the arrows.

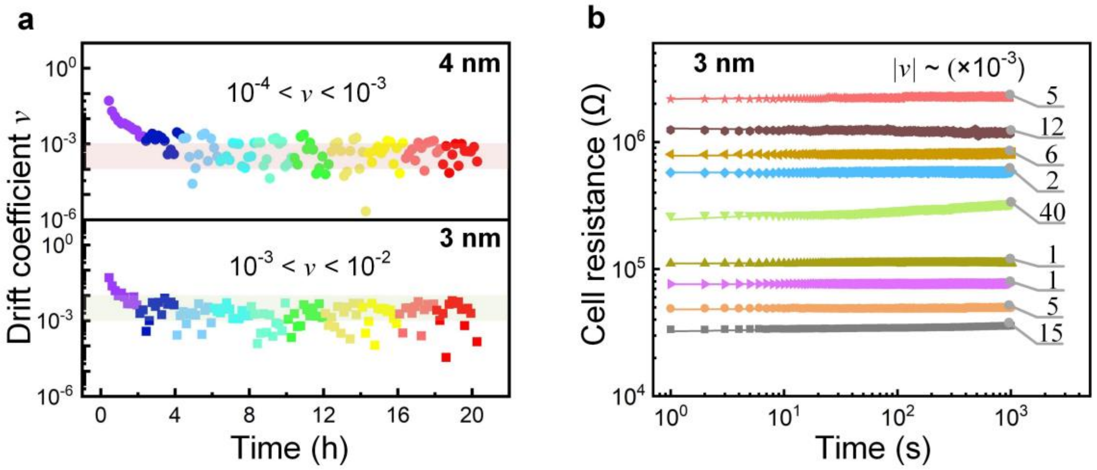

Figure S8. Larger resistance drift of the 3 nm-thick Sb films. (a) Resistance drift coefficient (sub *v*) as a function of time for the 3 nm-thick Sb film, with sub *v* mostly locating in the range of 10^{-3} to 10^{-2} after aging for ~4-20 h. For comparison, the sub *v* for the 4 nm-thick Sb film mainly situates from 10^{-3} to 10^{-4} after the same duration of aging. (b) Temporal evolution of 9-level resistant states realized by

iterative RESET operation for PCRAM device based on the 3 nm-thick Sb film. With the same device structure, reducing the film thickness from 4 to 3 nm increases the *v* with roughly one order of magnitude (see Figure 3F in the main text), ranging from 10^{-3} to 10^{-2}.

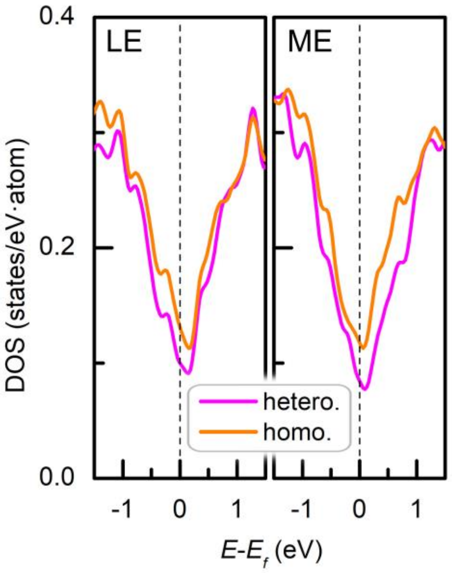

Figure S9. Partial electronic density of states (DOS) contributed by heteropolar (hetero.) and homopolar (homo.) bonds on the interfaces.

References

[1] Q. Hubert, C. Jahan, A. Toffoli, V. Delaye, D. Lafond, H. Grampeix, B. de Salvo, *IEEE Trans. Electron Dev.* **2013**, *60*, 2268.

[2] S. Kim, N. Sosa, M. BrightSky, D. Mori, W. Kim, Y. Zhu, K. Suu, C. Lam, in *2013 IEEE International Electron Devices Meeting*, **2013**, p. 30.7.1-30.7.4.

[3] M. He, D. He, H. Qian, Q. Lin, D. Wan, X. Cheng, M. Xu, H. Tong, X. Miao, *IEEE Electron Dev. Lett.* **2019**, *40*, 1595.

[4] S. Kim, N. Sosa, M. BrightSky, D. Mori, W. Kim, Y. Zhu, K. Suu, C. Lam, *IEEE Trans. Electron Dev.* **2016**, *63*, 3922.

[5] G. H. Oh, Y. L. Park, J. I. Lee, D. H. Im, J. S. Bae, D. H. Kim, D. H. Ahn, H. Horii, S. O. Park, H. S. Yoon, I. S. Park, Y. S. Ko, U.-I. Chung, J. T. Moon, in *2009 Symposium on VLSI Technology*, **2009**, pp. 220–221.

[6] I. S. Kim, S. L. Cho, D. H. Im, E. H. Cho, D. H. Kim, G. H. Oh, D. H. Ahn, S. O. Park, S. W. Nam, J. T. Moon, C. H. Chung, in *2010 Symposium on VLSI Technology*, **2010**, pp. 203–204.

[7] W. J. Wang, D. Loke, L. T. Law, L. P. Shi, R. Zhao, M. H. Li, L. L. Chen, H. X. Yang, Y. C. Yeo, A. O. Adeyeye, T. C. Chong, A. L. Lacaita, in *2012 International Electron Devices Meeting*, **2012**, p. 31.3.1-31.3.4.

[8] J. Li, B. Luan, T. H. Hsu, Y. Zhu, G. Martyna, D. Newns, H. Y. Cheng, S. Raoux, H. L. Lung, C. Lam, in *2011 International Electron Devices Meeting*, **2011**, p. 12.5.1-12.5.4.

[9] M. Mitra, Y. Jung, D. S. Gianola, R. Agarwal, *Appl. Phys. Lett.* **2010**, *96*, 222111.

[10] Y.-G. Liu, Y.-F. Chen, D.-L. Cai, Y.-Y. Lu, L. Wu, S. Yan, Y. Li, J.-J. Lu, L. Yu, Z.-T. Song, *Semicond. Sci. Technol.* **2019**, *34*, 105019.

[11] C. Li, C. Hu, J. Wang, X. Yu, Z. Yang, J. Liu, Y. Li, C. Bi, X. Zhou, W. Zheng, *J. Mater. Chem. C* **2018**, *6*, 3387.

[12] M. Wimmer, M. Kaes, C. Dellen, M. Salinga, *Front. Phys.* **2014**, *2*, 75.

[13] G. Betti Beneventi, L. Perniola, V. Sousa, E. Gourvest, S. Maitrejean, J. C. Bastien, A. Bastard, B. Hyot, A. Fargeix, C. Jahan, J. F. Nodin, A. Persico, A. Fantini, D. Blachier, A. Toffoli, S. Loubriat, A. Roule, S. Lhostis, H. Feldis, G. Reimbold, T. Billon, B. De Salvo, L. Larcher, P. Pavan, D. Bensahel, P. Mazoyer, R. Annunziata, P. Zuliani, F. Boulanger, *Solid-State Electron.* **2011**, *65–66*, 197.

[14] P. Noé, C. Sabbione, N. Castellani, G. Veux, G. Navarro, V. Sousa, F. Hippert, F. d'Acapito, *J. Phys. D: Appl. Phys.* **2015**, *49*, 035305.

[15] J. Luckas, A. Piarristeguy, G. Bruns, P. Jost, S. Grothe, R. M. Schmidt, C. Longeaud, M. Wuttig, *J. Appl. Phys.* **2013**, *113*, 023704.

[16] W. W. Koelmans, A. Sebastian, V. P. Jonnalagadda, D. Krebs, L. Dellmann, E. Eleftheriou, *Nat. Commun.* **2015**, *6*, 8181.

[17] K. Ding, J. Wang, Y. Zhou, H. Tian, L. Lu, R. Mazzarello, C. Jia, W. Zhang, F. Rao, E. Ma, *Science* **2019**, *366*, 210.

[18] M. Salinga, B. Kersting, I. Ronneberger, V. P. Jonnalagadda, X. T. Vu, M. L. Gallo, I. Giannopoulos, O. Cojocaru-Mirédin, R. Mazzarello, A. Sebastian, *Nat. Mater.* **2018**, *17,* 681.

[19] F. Jiao, B. Chen, K. Ding, K. Li, L. Wang, X. Zeng, F. Rao, *Appl. Mater. Today* **2020**, *20*, 100641.

[20] B. Kersting, V. Ovuka, V. P. Jonnalagadda, M. Sousa, V. Bragaglia, S. G. Sarwat, M. Le Gallo, M. Salinga, A. Sebastian, *Sci. Rep.* **2020**, *10*, 8248.

[21] J. Huang, B. Chen, G. Sha, H. Gong, T. Song, K. Ding, F. Rao, *Nano Lett.* **2023**, *23*, 2362.
